# Block Cipher's Substitution Box Generation Based on Natural Randomness in Underwater Acoustics and Knight's Tour Chain

**DOI:** 10.1155/2022/8338508

**Published:** 2022-05-20

**Authors:** Muhammad Fahad Khan, Khalid Saleem, Tariq Shah, Mohammad Mazyad Hazzazi, Ismail Bahkali, Piyush Kumar Shukla

**Affiliations:** ^1^Department of Computer Science, Quaid-i-Azam University, Islamabad, Pakistan; ^2^Department of Software Engineering, Foundation University Islamabad, Islamabad, Pakistan; ^3^Department of Mathematics, Quaid-i-Azam University, Islamabad, Pakistan; ^4^Department of Mathematics, College of Science, King Khalid University, Abha, Saudi Arabia; ^5^Department of Information Sciences, King Abdulaziz University Jeddah, Jeddah 21589, Saudi Arabia; ^6^Department of Computer Science & Engineering, University Institute of Technology, Rajiv Gandhi Proudyogiki Vishwavidyalaya, Bhopal, Madhya Pradesh, India

## Abstract

The protection of confidential information is a global issue, and block encryption algorithms are the most reliable option for securing data. The famous information theorist, Claude Shannon, has given two desirable characteristics that should exist in a strong cipher which are substitution and permutation in their fundamental research on “Communication Theory of Secrecy Systems.” block ciphers strictly follow the substitution and permutation principle in an iterative manner to generate a ciphertext. The actual strength of the block ciphers against several attacks is entirely based on its substitution characteristic, which is gained by using the substitution box (S-box). In the current literature, algebraic structure-based and chaos-based techniques are highly used for the construction of S-boxes because both these techniques have favourable features for S-box construction but also various attacks of these techniques have been identified including SAT solver, linear and differential attacks, Gröbner-based attacks, XSL attacks, interpolation attacks, XL-based attacks, finite precision effect, chaotic systems degradation, predictability, weak randomness, chaotic discontinuity, and limited control parameters. The main objective of this research is to design a novel technique for the dynamic generation of S-boxes that are safe against the cryptanalysis techniques of algebraic structure-based and chaos-based approaches. True randomness has been universally recognized as the ideal method for cipher primitives design because true random numbers are unpredictable, irreversible, and unreproducible. The biggest challenge we faced during this research was how can we generate the true random numbers and how can true random numbers utilized for strengthening the S-box construction technique. The basic concept of the proposed technique is the extraction of true random bits from underwater acoustic waves and to design a novel technique for the dynamic generation of S-boxes using the chain of knight's tour. Rather than algebraic structure- and chaos-based techniques, our proposed technique depends on inevitable high-quality randomness which exists in underwater acoustics waves. The proposed method satisfies all standard evaluation tests of S-boxes construction and true random numbers generation. Two million bits have been analyzed using the NIST randomness test suite, and the results show that underwater sound waves are an impeccable entropy source for true randomness. Additionally, our dynamically generated S-boxes have better or equal strength, over the latest published S-boxes (2020 to 2021). According to our knowledge first time, this type of research has been conducted, in which natural randomness of underwater acoustic waves has been used for the construction of block cipher's substitution box.

## 1. Introduction

Information security is the protection of secret data from illegal access, disclosure, inspection, destruction, disruption, and modification. The protection of confidential information is a global issue and block encryption algorithms are the most reliable option [1]. Block cipher is a branch of deterministic algorithm that works on the static length of bits, which is called block. Block cipher algorithms split the plaintext into various blocks of size *k*, to generate the same number of encrypted blocks of size *n*. Block ciphers encrypt one block at a time and the size of the output block is always equal to the input block and the transformation from input block to output block is done through the key whitening operation. Block cipher merged the confusion-diffusion primitives iteratively using a round function to generate an encrypted text. AES, DES, GOST, and BLOWFISH are the most prominent block ciphers of the industry that used the same strategy. For the block encryption algorithms such as AES, GOST, BLOWFISH, DES, and linear-differential attacks are the most powerful attacks [2–6]. In the differential attack, the basic purpose is to detect the sequential patterns from the encrypted text and for this purpose, the attacker tries to apply a specific set of inputs to trace the change in output. In the linear attack, the basic purpose is to find the linear relation among the plain text, cipher text with the corresponding keys. The responsibility to create a randomized relation among ciphertext and the key is on the confusion component; also, the confusion component is totally responsible to provide resistance against the linear and differential attacks [1–11]. Block cipher's confusion component is generally known as substitution (S-box) which transforms *k* input bits into *m* output bits through *S*:{0,1}^*k*^⟶{0,1}^*m*^, transforms vector *z* = [*z*_*n*-1_, *z*_*n*-2_, *z*_*n*-3_ … *z*0] into output vector *k* = [*k*_*n*-1_, *k*_*n*-2_, *k*_*n*-3_,…, *k*_0_].

As S-box is the only nonlinear primitive of block cipher, so the block cipher strength depends on its design. Cipher designers used various approaches to construct good quality S-boxes. Chaos-based and algebraic structure-based techniques are highly used for the construction of S-boxes. Chaos-based and algebraic structure-based techniques have favourable features for S-box construction, but many cryptanalysis of these techniques have been identified in the current literature. These cryptanalysis are described in Section 3. Underwater acoustic is generated by a diverse nature of sound sources such as underwater volcanoes, snapping shrimp, reverberation, vibrating objects, breaking waves, marine life, man-made sources, rain, geological activities, scattering waves, reflection waves, random motion of water molecules, lightning strikes, ice cracking, earthquake, compression, and decompression of water molecules [12–22]. Due to these diverse natures of sound sources, inevitably high-quality randomness exists in the amplitude characteristic of the underwater acoustics, which was our main source of inspiration because true randomness has been universally recognized as the ideal primitive for cryptography. True random numbers (TRNs) are unpredictable, irreversible, and unreproducible that is why cipher researchers endorsed the true random number for cryptographic primitives design [23–30].

The main idea of this research paper is extraction of true random bits from underwater acoustic waves and to design a novel technique for the dynamic generation of cryptographic S-boxes using the chain of knight's tour. The main benefit of our approach is that the proposed technique depends on the natural randomness of underwater acoustic waves for the construction of S-boxes and that's why various existing chaos and algebraic structure-based attacks are bypassed for our proposed technique.

The rest of the paper is arranged as follows.  Section 2 presents our main contribution. Section 3 shows the potential cryptanalysis and attacks.  Section 4 describes the proposed methodology for the dynamic generation of strong S-boxes.  Section 5 presents results and discussion.  Section 6 shows the conclusion.

## 2. Contribution

- A novel technique is proposed based on combination selection, for the generation of true random numbers from the randomness which exists in the amplitude property of underwater acoustics. As an assessment, two million bits have been analyzed using the NIST randomness test suite, and results show that underwater acoustic waves are an impeccable entropy source for TRNG.
- Knight's tour-based, a novel technique is proposed, for the dynamic generation of S-boxes and as a result attacks of algebraic- and chaos-based techniques are not applicable and irrelevant for our proposed technique.
- According to our knowledge first time, this type of research has been conducted, in which natural randomness of underwater acoustic waves has been used for the construction of block cipher's substitution box.

## 3. Potential Attacks of Existing S-Box Designs

As we said before, chaos-based and algebraic structures-based techniques are widely used for the construction of Shannon's confusion primitive but many attacks of these techniques have been identified in the current literature including Gröbner-based attacks [2–8], SAT solver [9–11, 31–35], linear and differential attacks [36–50], XSL attacks [51–55], interpolation attacks [51, 56–58], XL-based attacks [59–61], finite precision effect [62–67], chaotic systems degradation [61–63, 68, 69], predictability [70, 71], weak randomness [62, 63, 65, 66, 72–77], chaotic discontinuity [65–67, 72, 73], and limited control parameters [78–81].

The main objective of this research is to design a novel technique for the dynamic generation of S-boxes that are safe against the attacks of algebraic structure-based and chaos-based techniques. Rather than algebraic structure- and chaos-based techniques, our proposed technique depends on inevitable high-quality randomness which exists in underwater acoustics waves. The basic concept of the proposed technique is the extraction of true random bits from underwater acoustic waves and to design a novel technique for the dynamic generation of S-boxes using the chain of knight's tour.

## 4. Proposed Methodology

**Figure 1:**
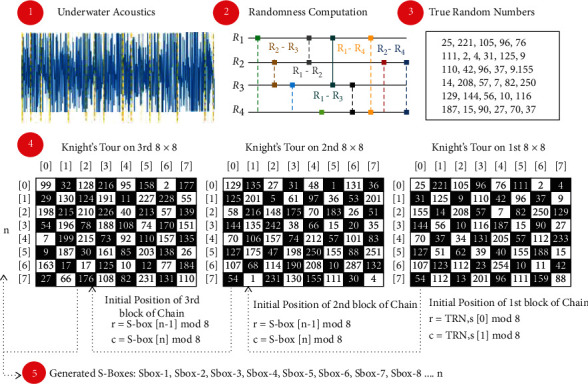
Architecture diagram of the proposed system.

The proposed method consists of two phases, the first phase is true random numbers generation based on underwater acoustics and the second phase is dynamic generation of S-boxes based on Knight's tour chain. Architecture diagram of the proposed system is depicted in Figure 1.

### 4.1. True Random Numbers Generation Based on Underwater Acoustics

**Figure 2:**
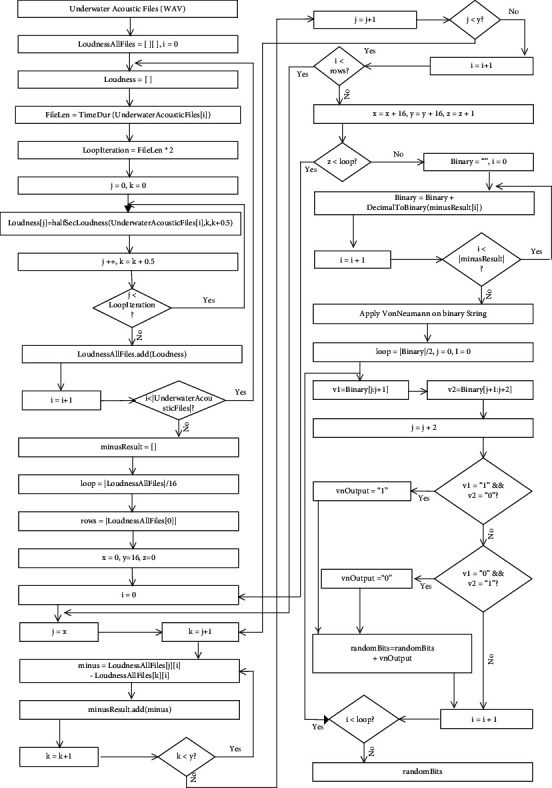
Flowchart of TRNG-based underwater acoustics.

**Figure 3:**
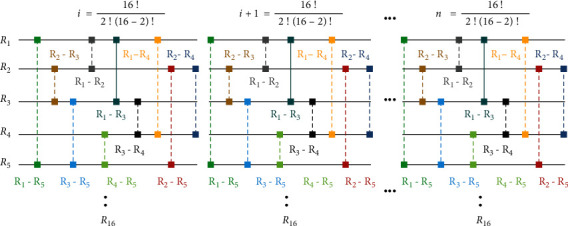
Computation of amplitude differences.

In this phase, first of all, long-term underwater acoustics recordings were acquired from the doi based dataset of the Australian Antarctic Data Centre (AADC) [82]. In the dataset, the average duration of each recording is sixty minutes. Out of thousands of long-term underwater acoustic recordings, we randomly selected the 96 long-term underwater acoustic recordings but proposed technique can take any multiple of 16 files as entropy sources. Secondly, these recordings are divided into blocks of size 16 and then, the amplitude difference of every 0.5 sec is calculated. Due to the diverse nature of sound sources, the difference of each amplitude with other amplitudes is random, and this was our main source of inspiration. Other characteristics of underwater sound like frequency and timber contain low-quality randomness that is why we chose the amplitude characteristic for this research. To calculate the amplitude differences, we used the combination selection strategy by using *n*!/*r*! (*n*–*r*)! . In our case, the value of the *n* is 16 and the value of *r* is 2. The entire step-by-step process of this phase from underwater acoustic files input to the random bits generation is represented in the flowchart of Figure 2. The amplitude differences calculation step is depicted in Figure 3, and here, long-term underwater acoustic recording represented as *R*_1_, *R*_2_,… *R*_16_. Two million bits have been analyzed using the NIST randomness test suite and shown in the Table 1, and results of the NIST tests show that underwater acoustics waves are an impeccable entropy source for true randomness. There are many random extractors based on hash functions, machine learning, chaos machine, physical unclonable functions, and probabilistic methods but among all these types of random extractors. Von Neumann random extractor is the simplest and fastest method that is why we chose Von Neumann random extractor as the postprocessing method.

### 4.2. Dynamic Generation of S-Boxes Based on Knight's Tour Chain

**Figure 4:**
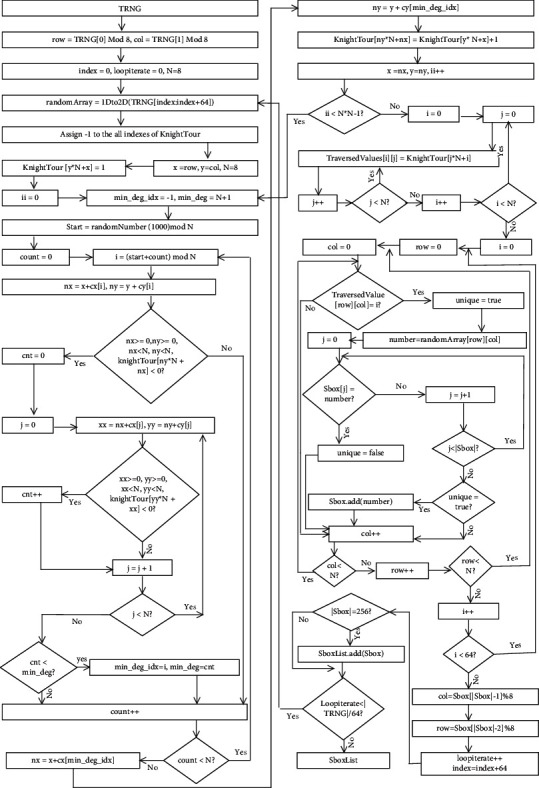
Flowchart of dynamic S-boxes generation based on knight's tour chain.

**Figure 5:**
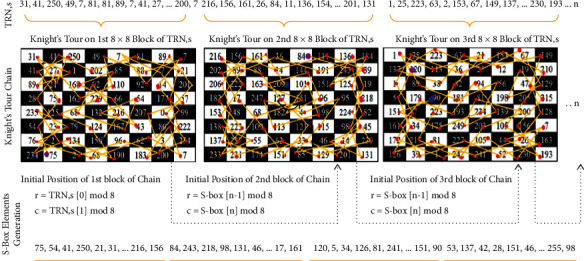
Dynamic generation of S-boxes based on knight's tour chain.

The knight's tour is more than a 1400-year-old puzzle game whose objective is to discover the legal moves on the chessboard in the way that it explores every cell only once, and in our proposed methodology, we utilized the chain of 8 × 8 knight's tour for the generation of S-boxes. First of all, true random numbers are acquired and divided into blocks of 64 length size, and then, each 64 length block is converted into the 8 × 8 chessboard matrix. Based on the knight tour rules, we traversed each element of the chessboard matrix; however, only unique elements are considered for S-box elements, and a similar procedure is repeated for coming chessboards until the completion of required length of the S-box. Initial position of the first block of the knight's tour chain is calculated through *r* = TRNG [0] mod 8, *c* = TRNG [1] mod 8, and the initial positions of other knights' tour chains are dependent on the second last and the last element of the S-box, which are calculated through *r* = S-box [*n* − 1] mod 8, *c* = S-box [*n*] mod 8. The entire step-by-step process of this phase from true random numbers input to dynamic S-boxes generation is represented in the flowchart of Figure 4. This phase is depicted in the following Figure 5. The reverse S-box algorithm is shown in the following (Algorithm 1). From the dynamically generated S-boxes stream, we picked two S-boxes randomly as sample which are shown in Tables 2 and 3, and their reverse S-boxes are also shown in Tables 4 and 5, respectively. The maximum nonlinearity score of our sample S-boxes is higher or equal to the recently published S-boxes (from 2020 to 2021).

## 5. Results and Evaluation

In the results and evaluation section, our proposed S-boxes are evaluated by standard S-box evaluation criteria which includes nonlinearity score, bit independence criterion, linear approximation probability, strict avalanche criterion, and differential approximation probability.

### 5.1. Nonlinearity

Among all cryptographic properties, nonlinearity is the most important one. The main purpose of S-box is to gain nonlinear change from secret message to the ciphered message. For a strong encryption scheme, the mapping between input and output in an S-box must be nonlinear. The nonlinearity of the cryptographic algorithm is represented by the nonlinearity score. Nonlinearity is defined as the smallest difference of the Boolean function to the bunch of affine functions. The nonlinearity score determine the total number of bits altered to get the closest affine function in the Boolean truth table. It calculates the distance between the set of all affine functions and Boolean function. When the initial distance is obtained, the nearest affine function is achieved by inverting the bit values in the truth table of the Boolean function. By using walsh spectrum, the nonlinearity of the Boolean function is computed through [46]:

(1) $$\begin{array}{c} N_{g}=2^{n-1}\left(1-2^{-n}\underset{\phi \varepsilon \text{ GF}\left(2^{n}\right)}{\max}\left|S\left(g\right)\left(\phi \right)\right|\right), \end{array}$$

*S*_(*g*)_ (*φ* is defined as

(2) $$\begin{array}{c} \;S_{\left(g\right)}\left(\varphi \right)=\sum_{\phi \in \text{ GF}\left(2^{n}\right)}{\left(-1\right)}^{g\left(x\right)⊕x.\phi }, \end{array}$$

where *φ* is a n-bit vector and *φ* ∈ GF(2^*n*^).*x*.*φ* represents the bit-wise dot product of *x* and *φ*:

(3) $$\begin{array}{c} x.\varphi =x1⊕\varphi 1+x2⊕\varphi 2\cdots +\text{xn}⊕\varphi n. \end{array}$$

S-box having high nonlinearity creates difficult for attacker to perform linear cryptanalysis. The maximum nonlinearity scores of our proposed Sbox-1 and Sbox-2 are 110 and 110, respectively, which is higher or equal to the recently published S-boxes. Detailed comparative analysis is shown in Table 6.

### 5.2. Strict Avalanche Criteria (SAC)

Strict avalanche criteria is the another crucial property for evaluating and according to SAC, if a single input bit is altered, all output bits will shift with probability of 1/2. SAC examined the effects of avalanche affects in encryption scheme. The modification at the input series induces a significant change in output series. SAC computes the number of output bits altered caused by inverting a single bit of input. To make the system more reliable, the output vector needed to be deviate with half probability, when one bit of input is inverted. Dependency matrix is determined to evaluate the SAC property. For an S-box that satisfies SAC property, all values were close to the ideal value of 0.5 in its dependence matrix. Dependency matrix offsets computed through equation (4) [46]. The SAC results of S-box-1 and S-box-2 are shown in Tables 7 and 8 and scores of our S-box-1 and S-box-2 are 0.495 and 0.50, respectively, which are the ideal scores for the secure S-boxes.

(4) $$\begin{array}{c} S\left(g\right)=\frac{1}{n^{2}}\sum_{1\leq r\leq n}\sum_{1\leq w\leq n}\;\left|\frac{1}{2}-\text{Qr},w\left(g\right)\right|, \end{array}$$

where

(5) $$\begin{array}{c} Q_{r,w}\left(g\right)=2^{-n}\sum_{x\varepsilon B^{n}}\text{gw}\left(x\right)⊕\text{gw}\left(x\;⊕\;e_{r}\right), \end{array}$$

*e*_*r*_=[*θr*, 1*θr*, 2…*θr*, *n*]^*T*^,^T^ is the transpose of matrix *θ*_*r*,*w*_=0, *r* ≠ *w* or. *θ*_*r*,*w*_= 1,  *r*=*w*.

### 5.3. BIT Independent Criterion (BIC)

BIC requires that all avalanche variables for a given set of avalanche vectors must be pair-wise independent. By modifying the input bits, BIC is used to study the behaviour of the output bits. When the output bits behave independent of one another, the S-box holds the BIC property. If any single input bit *i* is inverted, BIC states that output bits *j* and *k* will alter independently. This will enhance the effectiveness of confusion function. The coefficient of correlation is used to determine the independence among pair of avalanche variables. High bit independence is required to make system design incomprehensible. The bit independence of the *j*^th^ and *k*^th^ bits of *B*^*ei*^ is [46]: in Tables 9 and 10, we can see that our randomly picked S-box-1 and S-box-2 fully fill the BIT independent criterion.

(6) $$\begin{array}{c} \text{BIC}\left(b_{j},\;b_{k}\right)=\;\underset{1\leq i\leq n}{\max}\left|\text{corr}\left(b_{j}^{\text{ei}},b_{j}^{\text{ei}}\right)\right|. \end{array}$$

S-box function (*h*) is described as: *h*: {0, 1}^*n*^ ⟶{0, 1}^*n*^

BIC parameter for the S-box function is expressed as

(7) $$\begin{array}{c} \text{BIC}\left(h\right)=\;\underset{1\leq j\;,\;k\leq n}{\max}\text{BIC}\left(b_{j},\;b_{k}\right). \end{array}$$

The change in output bits is a crucial parameter in determining the cipher's strength. When the changes in output bits contrast with the input bit sequence shows sufficient independence, the mapping technique will be difficult to understand.

### 5.4. Linear Approximation Probability (LP)

LP is the cryptographic property which measures the resistance of S-box against the linear attacks. LP analysis intends to measure the maximum imbalance of the event. LP is measured by determining the total number of coincident input bits with the output bits. The input bits uniformity must be identical to the output bits. Each input bit is individually evaluated, and its results are tested in the output bits. *γ*1 and *γ*2 masks are selected randomly to determine the mask of all output and input values. The mathematical expression of determining linear approximation probability is as follows [46]: the maximum LP of S-box-1 and S-box-2 is 0.125, which is also satisfies LP criteria.

(8) $$\begin{array}{c} {\text{LP}}_{f}=\max_{\gamma 1,\gamma 2\neq 0}\left|\frac{\left\{xɛX|x.\gamma 1=S\left(x\right).\gamma 2\right\}}{2^{n}}-\frac{1}{2}\right|, \end{array}$$

where *γ*1 and *γ*2  represents the input and output mask in the above expression. Linear approximation probability is calculated by using these masks. *X* represents the set of all possible inputs and 2^n^ is the total number of elements in the set. S-box with low LP value is robust enough against different linear approximation attacks.

### 5.5. Differential Approximation Probability (DP)

The resistance of S-box to the differential attacks is assessed through the DP. DP is the probability of particular change in output bits caused by the change in input bits. An S-box must possess differential uniformity which means that each input differential is connected to the specific output differential. The XOR values of all output must have equal probability to the XOR values of all input. The differential uniformity is measured by given expression [46]:

(9) $$\begin{array}{c} \text{DP}\left(\Delta x⟶\Delta y\right)=\left[\frac{\#\left\{xɛX|\left(S\left(x\right)⊕S\left(x⊕\Delta x\right)=\Delta y\right)\right\}}{2^{n}}\right], \end{array}$$

where *X* represents the set of all possible input values and 2^n^ is the total number of elements in set. The maximum differential probability value a system could achieve is 4/256. The lowest value of DP means the high security of the S-box against differential approximation attacks. In Tables 11 and 12, we can see that our randomly picked S-box-1 and S-box-2 fully fill the DP criterion.

## 6. Conclusion

The protection of confidential information is a global issue, and block encryption algorithms are the most reliable option. The actual strength of the block encryption algorithms against several attacks is entirely dependent on S-boxes. Currently in the literature, algebraic structure-based and chaos-based techniques are highly used for the construction of S-boxes because both these techniques have favourable features for S-box construction, but many attacks of these techniques have been identified. In this paper, we purposed a novel technique for the dynamic generation of S-boxes that is safe against the existing attacks of algebraic structure-based and chaos-based techniques. True randomness has been universally recognized as the ideal method for security primitive because true random numbers are unpredictable, irreversible, and unreproducible. Rather than algebraic structure- and chaos-based techniques, our proposed technique depends on inevitable high-quality randomness which exists in underwater acoustics waves. According to our knowledge first time, this type of research has been conducted, in which natural randomness of underwater acoustic waves and knight's tour problem has been used for the generation of block cipher's substitution box. The proposed method satisfies all standard evaluation tests of S-boxes construction and true random numbers generation. Additionally, our dynamically generated S-boxes have better or equal strength, over the latest published S-boxes (2020 to 2021). In the future, we will extend this research for automatic key generation and optimization using knight's tour.

## Figures and Tables

**Algorithm 1:**
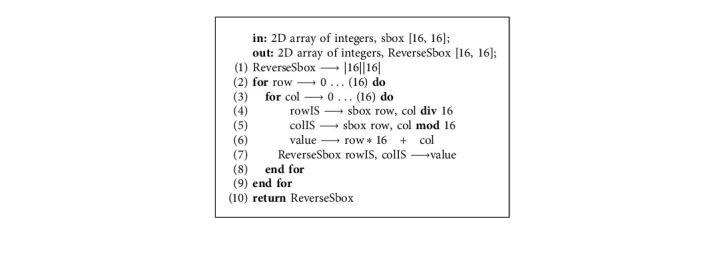
ReverseSbox (S-box).

**Table 1:** Results of NIST randomness test suite.

Type of test	*P* value			Conclusion
01. Frequency test (monobit)	0.8461758819031635			Random
02. Frequency test within a block	0.5166228701210154			Random
03. Run test	0.2609970420138874			Random
04. Longest run of ones in a block	0.34640251063536204			Random
05. Binary matrix rank test	0.09949346113140206			Random
06. Discrete Fourier transform (spectral) test	0.832838521091328			Random
07. Nonoverlapping template matching test	0.22184797295460632			Random
08. Overlapping template matching test	0.16413619193258017			Random
09. Maurer's universal statistical test	0.4051810932845413			Random
10. Linear complexity test	0.4394606534399792			Random
11. Serial test:	0.5703210920746249			Random
	0.5412977586951687			Random
12. Approximate entropy test	0.013704869478928823			Random
13. Cummulative sums (forward) test	0.9081561792752144			Random
14. Cummulative sums (reverse) test	0.7420961383854099			Random
15. Random excursions test:	State	Chi squared	*P* value	Conclusion
	−4	3.1940558536339703	0.6700965356355721	Random
	−3	8.322704313725493	0.13932469392722086	Random
	−2	2.215379649802308	0.8186113923928053	Random
	−1	10.373856209150327	0.06530927491189864	Random
	+1	5.49281045751634	0.3587348843928551	Random
	+2	1.978633099330267	0.8520935894148005	Random
	+3	0.872903529411762	0.9721522155809542	Random
	+4	2.2030722493078865	0.8203920781040164	Random
16. Random excursions variant test:	State	Counts	*P* value	Conclusion
	−9.0	1743	0.3503620748973999	Random
	−8.0	1791	0.22313138786599762	Random
	−7.0	1861	0.09700096546916874	Random
	−6.0	1837	0.09426256021374013	Random
	−5.0	1755	0.17515792247265105	Random
	−4.0	1675	0.3218141454622986	Random
	−3.0	1588	0.6391395377015844	Random
	−2.0	1605	0.43375610043914314	Random
	−1.0	1572	0.4476990724652935	Random
	+1.0	1601	0.19931513588782468	Random
	+2.0	1629	0.30147752489003166	Random
	+3.0	1609	0.523032983174088	Random
	+4.0	1609	0.589348273539888	Random
	+5.0	1662	0.426374068680618	Random
	+6.0	1783	0.1678955379041649	Random
	+7.0	1876	0.0827802734496795	Random
	+8.0	1869	0.1135773370125223	Random
	+9.0	1818	0.20668955769990105	Random

**Table 2:** Substitution box 1.

106	220	5	24	1	124	20	104	96	88	240	170	9	115	246	86
197	230	174	155	76	185	175	31	142	103	239	122	40	113	208	228
78	21	218	29	110	85	43	70	27	120	66	28	189	126	36	232
138	165	234	16	243	23	160	235	97	48	90	101	98	250	6	45
38	73	141	53	81	71	203	206	2	135	252	111	145	92	238	63
130	186	180	123	192	4	251	89	196	84	58	143	32	59	82	198
112	224	247	64	177	178	148	184	233	200	222	107	105	195	201	187
154	236	163	109	219	254	137	210	241	204	212	139	34	248	249	74
202	253	52	47	226	19	12	3	114	207	118	171	91	193	217	144
169	237	13	57	131	30	121	95	33	14	199	119	146	100	166	182
255	72	215	209	188	77	99	35	116	242	18	87	132	102	158	152
150	62	211	55	164	80	162	125	225	133	183	117	179	51	205	60
65	8	15	213	69	223	41	54	176	46	244	194	0	156	172	39
56	161	227	147	93	129	67	168	221	245	25	136	17	214	128	167
61	229	22	11	153	94	149	151	50	216	49	159	37	10	127	7
26	83	44	134	42	231	79	75	68	108	173	157	140	191	181	190

**Table 3:** Substitution box 2.

254	240	187	11	151	155	153	100	103	201	144	0	72	14	158	63
180	209	138	2	169	27	60	186	21	97	52	109	251	248	95	19
124	71	10	107	58	210	26	203	90	168	121	250	66	226	50	104
176	46	65	93	6	183	245	134	86	216	7	44	238	207	16	110
202	99	17	165	217	167	80	55	128	82	75	200	40	182	147	174
196	156	120	192	116	136	164	188	48	5	152	166	33	62	230	137
12	9	102	61	223	54	159	34	59	246	195	213	170	51	253	229
126	122	140	241	98	77	237	179	47	191	30	130	118	185	224	243
45	36	227	149	106	239	68	221	189	219	150	108	13	161	154	112
242	172	23	178	135	131	160	231	129	244	31	255	173	39	233	205
198	89	20	18	215	8	249	139	181	212	53	163	157	127	208	64
105	85	142	184	145	29	37	175	111	125	222	4	117	232	76	87
84	35	42	123	49	235	24	92	101	91	204	194	79	133	96	32
15	69	67	146	190	88	83	1	141	119	177	234	132	38	74	236
252	3	28	78	22	220	57	43	211	225	199	41	94	197	143	70
206	73	162	56	25	228	218	81	115	114	171	113	148	193	247	214

**Table 4:** Reverse S-box 1.

204	4	72	135	85	2	62	239	193	12	237	227	134	146	153	194
51	220	170	133	6	33	226	53	3	218	240	40	43	35	149	23
92	152	124	167	46	236	64	207	28	198	244	38	242	63	201	131
57	234	232	189	130	67	199	179	208	147	90	93	191	224	177	79
99	192	42	214	248	196	39	69	161	65	127	247	20	165	32	246
181	68	94	241	89	37	15	171	9	87	58	140	77	212	229	151
8	56	60	166	157	59	173	25	7	108	0	107	249	115	36	75
96	29	136	13	168	187	138	155	41	150	27	83	5	183	45	238
222	213	80	148	172	185	243	73	219	118	48	123	252	66	24	91
143	76	156	211	102	230	176	231	175	228	112	19	205	251	174	235
54	209	182	114	180	49	158	223	215	144	11	139	206	250	18	22
200	100	101	188	82	254	159	186	103	21	81	111	164	44	255	253
84	141	203	109	88	16	95	154	105	110	128	70	121	190	71	137
30	163	119	178	122	195	221	162	233	142	34	116	1	216	106	197
97	184	132	210	31	225	17	245	47	104	50	55	113	145	78	26
10	120	169	52	202	217	14	98	125	126	61	86	74	129	117	160

**Table 5:** Reverse S-box 2.

11	215	19	225	187	89	52	58	165	97	34	3	96	140	13	208
62	66	163	31	162	24	228	146	198	244	38	21	226	181	122	154
207	92	103	193	129	182	221	157	76	235	194	231	59	128	49	120
88	196	46	109	26	170	101	71	243	230	36	104	22	99	93	15
175	50	44	210	134	209	239	33	12	241	222	74	190	117	227	204
70	247	73	214	192	177	56	191	213	161	40	201	199	51	236	30
206	25	116	65	7	200	98	8	47	176	132	35	139	27	63	184
143	251	249	248	84	188	124	217	82	42	113	195	32	185	112	173
72	152	123	149	220	205	55	148	85	95	18	167	114	216	178	238
10	180	211	78	252	131	138	4	90	6	142	5	81	172	14	102
150	141	242	171	86	67	91	69	41	20	108	250	145	156	79	183
48	218	147	119	16	168	77	53	179	125	23	2	87	136	212	121
83	253	203	106	80	237	160	234	75	9	64	39	202	159	240	61
174	17	37	232	169	107	255	164	57	68	246	137	229	135	186	100
126	233	45	130	245	111	94	151	189	158	219	197	223	118	60	133
1	115	144	127	153	54	105	254	29	166	43	28	224	110	0	155

**Table 6:** Nonlinearity of state-of-the-art S-boxes.

Recently published S-boxes	Maximum nonlinearity achieved
[83], 2021	−108
[85], 2021	−110
[87], 2021	−108
[89], 2020	−108
[91], 2020	−110
[93], 2020	−108
[95], 2020	−108
[97], 2020	−108
[99], 2020	−104
[99], 2020	−108
[98], 2020	−106
[101], 2020	−108
[102], 2020	−110
[104], 2021	−108
[105], 2021	−110
[84], 2021	−110
[86], 2021	−110
[88], 2021	−108
[90], 2021	−110
[92], 2020	−102
[94], 2020	−107
[96], 2020	−104
[98], 2020	−106
[100], 2020	−105
[101], 2020	−106
[95], 2020	−108
[93], 2020	−108
[103], 2020	−108
[104], 2021	−108
[106], 2021	−108

**Table 7:** SAC results of S-box-1.

0.500000	0.562500	0.468750	0.453125	0.500000	0.421875	0.453125	0.500000
0.437500	0.515625	0.468750	0.468750	0.515625	0.500000	0.546875	0.437500
0.468750	0.546875	0.484375	0.515625	0.500000	0.531250	0.546875	0.500000
0.453125	0.500000	0.500000	0.500000	0.484375	0.453125	0.515625	0.546875
0.468750	0.562500	0.500000	0.500000	0.484375	0.437500	0.484375	0.500000
0.406250	0.546875	0.593750	0.484375	0.453125	0.390625	0.531250	0.500000
0.437500	0.484375	0.578125	0.453125	0.515625	0.546875	0.437500	0.484375
0.546875	0.515625	0.531250	0.500000	0.562500	0.437500	0.515625	0.515625

**Table 8:** SAC results of S-box-2.

0.531250	0.546875	0.546875	0.468750	0.421875	0.437500	0.546875	0.500000
0.546875	0.531250	0.406250	0.484375	0.562500	0.468750	0.484375	0.453125
0.515625	0.484375	0.500000	0.578125	0.640625	0.515625	0.546875	0.437500
0.562500	0.468750	0.453125	0.437500	0.500000	0.546875	0.546875	0.546875
0.593750	0.546875	0.531250	0.593750	0.500000	0.500000	0.468750	0.531250
0.500000	0.468750	0.531250	0.531250	0.437500	0.484375	0.484375	0.484375
0.484375	0.421875	0.546875	0.484375	0.437500	0.515625	0.515625	0.546875
0.500000	0.453125	0.578125	0.468750	0.562500	0.531250	0.562500	0.421875

**Table 9:** BIC independent matrix of S-box-1.

—	0.480469	0.484375	0.464844	0.509766	0.507812	0.517578	0.521484
0.480469	—	0.511719	0.513672	0.484375	0.486328	0.476562	0.494141
0.484375	0.511719	—	0.498047	0.507812	0.494141	0.503906	0.486328
0.464844	0.513672	0.498047	—	0.494141	0.505859	0.501953	0.496094
0.509766	0.484375	0.507812	0.494141	—	0.509766	0.480469	0.470703
0.507812	0.486328	0.494141	0.505859	0.509766	—	0.494141	0.498047
0.517578	0.476562	0.503906	0.501953	0.480469	0.494141	—	0.509766
0.521484	0.494141	0.486328	0.496094	0.470703	0.498047	0.509766	—

**Table 10:** BIC independent matrix of S-box-2.

—	0.501953	0.498047	0.501953	0.488281	0.529297	0.486328	0.484375
0.501953	—	0.500000	0.501953	0.484375	0.513672	0.466797	0.509766
0.498047	0.500000	—	0.507812	0.527344	0.474609	0.507812	0.486328
0.501953	0.501953	0.507812	—	0.519531	0.521484	0.494141	0.511719
0.488281	0.484375	0.527344	0.519531	—	0.523438	0.515625	0.521484
0.529297	0.513672	0.474609	0.521484	0.523438	—	0.478516	0.503906
0.486328	0.466797	0.507812	0.494141	0.515625	0.478516	—	0.519531
0.484375	0.509766	0.486328	0.511719	0.521484	0.503906	0.519531	—

**Table 11:** DP of S-box-1.

0.00000	0.02343	0.03125	0.03125	0.02343	0.02343	0.02343	0.02343	0.03125	0.02343	0.02343	0.02343	0.02343	0.02343	0.02343	0.03125
0.03125	0.03125	0.02343	0.03125	0.02343	0.02343	0.02343	0.03125	0.02343	0.02343	0.03125	0.02343	0.02343	0.02343	0.03125	0.03125
0.03125	0.03125	0.02343	0.02343	0.03125	0.02343	0.03125	0.039062	0.02343	0.02343	0.02343	0.02343	0.02343	0.02343	0.02343	0.03125
0.02343	0.03125	0.02343	0.02343	0.03125	0.02343	0.02343	0.02343	0.02343	0.02343	0.02343	0.02343	0.02343	0.02343	0.02343	0.03906
0.02343	0.02343	0.03125	0.02343	0.03125	0.03125	0.03125	0.02343	0.02343	0.02343	0.03125	0.03125	0.03125	0.02343	0.02343	0.02343
0.03125	0.02343	0.02343	0.02343	0.02343	0.02343	0.02343	0.02343	0.02343	0.03125	0.03125	0.03125	0.02343	0.03125	0.03125	0.03125
0.02343	0.03125	0.03125	0.02343	0.02343	0.02343	0.02343	0.03125	0.02343	0.02343	0.02343	0.02343	0.02343	0.03125	0.02343	0.02343
0.02343	0.03125	0.02343	0.03125	0.01562	0.03125	0.02343	0.02343	0.02343	0.02343	0.02343	0.02343	0.02343	0.01562	0.02343	0.02343
0.03125	0.02343	0.02343	0.02343	0.03125	0.02343	0.03125	0.01562	0.02343	0.02343	0.02343	0.03125	0.03906	0.03125	0.02343	0.03125
0.03125	0.02343	0.02343	0.03125	0.03125	0.02343	0.03125	0.02343	0.03125	0.02343	0.02343	0.02343	0.02343	0.02343	0.02343	0.02343
0.02343	0.02343	0.02343	0.02343	0.03125	0.02343	0.02343	0.03125	0.02343	0.02343	0.02343	0.02343	0.02343	0.02343	0.02343	0.02343
0.02343	0.03125	0.02343	0.02343	0.02343	0.02343	0.02343	0.02343	0.02343	0.02343	0.03125	0.02343	0.02343	0.02343	0.02343	0.02343
0.02343	0.02343	0.02343	0.03125	0.02343	0.02343	0.03125	0.03125	0.02343	0.03125	0.03125	0.02343	0.02343	0.02343	0.02343	0.03125
0.01562	0.02343	0.02343	0.02343	0.03125	0.02343	0.03125	0.02343	0.02343	0.03906	0.03125	0.02343	0.02343	0.03125	0.03125	0.02343
0.02343	0.02343	0.02343	0.03125	0.02343	0.02343	0.02343	0.02343	0.03125	0.02343	0.02343	0.03125	0.02343	0.03906	0.02343	0.02343
0.02343	0.02343	0.02343	0.02343	0.02343	0.02343	0.02343	0.02343	0.03125	0.02343	0.03125	0.01562	0.02343	0.02343	0.01562	0.02343

**Table 12:** DP of S-box-2.

0.00000	0.02343	0.02343	0.03125	0.02343	0.015625	0.02343	0.03125	0.03906	0.02343	0.03125	0.02343	0.03125	0.02343	0.02343	0.02343
0.02343	0.02343	0.02343	0.02343	0.03125	0.02343	0.02343	0.02343	0.03125	0.02343	0.02343	0.02343	0.03125	0.015625	0.02343	0.02343
0.02343	0.02343	0.02343	0.02343	0.02343	0.03125	0.02343	0.02343	0.02343	0.03125	0.02343	0.03125	0.02343	0.02343	0.02343	0.02343
0.02343	0.02343	0.03125	0.02343	0.02343	0.03125	0.02343	0.03125	0.02343	0.02343	0.03125	0.03125	0.03125	0.02343	0.02343	0.02343
0.03125	0.02343	0.02343	0.03906	0.03125	0.03125	0.02343	0.02343	0.02343	0.02343	0.02343	0.02343	0.02343	0.02343	0.02343	0.02343
0.03125	0.02343	0.02343	0.03125	0.02343	0.02343	0.02343	0.03125	0.02343	0.03125	0.03125	0.03125	0.03906	0.02343	0.03125	0.02343
0.02343	0.02343	0.02343	0.02343	0.03125	0.03125	0.03125	0.02343	0.02343	0.02343	0.02343	0.02343	0.03125	0.03125	0.02343	0.02343
0.02343	0.02343	0.02343	0.02343	0.03125	0.02343	0.02343	0.03125	0.03125	0.03125	0.02343	0.02343	0.03125	0.03125	0.03125	0.03125
0.02343	0.02343	0.03125	0.03125	0.03125	0.03125	0.03906	0.02343	0.03125	0.02343	0.02343	0.02343	0.03125	0.02343	0.02343	0.02343
0.02343	0.02343	0.02343	0.02343	0.02343	0.03125	0.02343	0.02343	0.02343	0.02343	0.03125	0.02343	0.03125	0.02343	0.02343	0.02343
0.02343	0.03125	0.02343	0.02343	0.02343	0.02343	0.02343	0.02343	0.02343	0.02343	0.03125	0.02343	0.03125	0.02343	0.015625	0.03125
0.02343	0.02343	0.02343	0.02343	0.02343	0.02343	0.02343	0.02343	0.02343	0.02343	0.02343	0.02343	0.03125	0.02343	0.02343	0.02343
0.02343	0.02343	0.02343	0.03125	0.03125	0.02343	0.02343	0.03125	0.03125	0.02343	0.03125	0.02343	0.02343	0.03125	0.02343	0.02343
0.02343	0.03125	0.02343	0.03125	0.02343	0.02343	0.02343	0.02343	0.03125	0.02343	0.03125	0.02343	0.02343	0.02343	0.02343	0.02343
0.02343	0.03125	0.02343	0.02343	0.02343	0.03125	0.02343	0.03125	0.03125	0.03906	0.02343	0.02343	0.02343	0.015625	0.015625	0.03125
0.02343	0.02343	0.02343	0.02343	0.03906	0.02343	0.03125	0.03125	0.03125	0.03125	0.02343	0.02343	0.02343	0.03125	0.02343	0.03906

## Data Availability

The datasets analyzed during the current study are available in the Australian Antarctic Data Centre repository at https://data.aad.gov.au/metadata/records/AAS_4102_longTermAcousticRecordings.
